# A population-based cohort study of HRT use and breast cancer in southern Sweden

**DOI:** 10.1054/bjoc.2001.1899

**Published:** 2001-09

**Authors:** H Olsson, A Bladström, C Ingvar, T R Möller

**Affiliations:** Department of Oncology, University Hospital, Lund, S-22185, Sweden; South Swedish Regional Tumour Registry, Lund, S-22185, Sweden; Department of Surgery, University Hospital, Lund, S-22185, Sweden

**Keywords:** hormone replacement therapy, tumour incidence, family history, present use, past use

## Abstract

The overall tumour incidence and breast cancer incidence related to hormone replacement therapy (HRT) were followed in a population-based cohort of 29 508 women, aged 25–65 when interviewed in 1990–92. By the end of the follow up in December 1999, there were 226 611 person-years of observation. A total of 1145 malignant tumours were recorded (expected 1166.6; SIR = 0.98, 95% CI 0.93–1.04). There was a small excess of breast cancer with 434 observed and 387.69 expected (SIR = 1.12, 95% CI 1.02–1.23). Among about 3 663 ever users of HRT, there was no increase in overall tumour incidence (SIR = 0.98, 95% CI 0.86–1.12) but a significant excess of breast cancer (SIR = 1.35, 95% CI 1.09–1.64) compared with never users (SIR = 1.07, 95% CI 0.96–1.19). Breast cancer increased with increasing duration of use and for 48–120 months use the SIR was 1.92 (95% CI 1.32–2.70). There was no significant interaction with family history of breast cancer although an independent additive effect was suggested between HRT use and family history. In a Cox regression model time to breast cancer in relation to duration of HRT use was analysed adjusting for age at menarche, age at menopause, age at first full term pregnancy, parity and age at diagnosis. A significantly higher risk was seen for longer duration of HRT use compared with never users. No increased risk is seen in women beyond 5 years after stopping HRT. There was no interaction between previous use of oral contraceptives and later HRT use. © 2001 Cancer Research Campaign http://www.bjcancer.com


BJOC 01-1899 674-677 20/8/01 3:05 pm Page 674

*British Journal of Cancer* (2001) **85**(5), 674–677
© 2001 Cancer Research Campaign
doi: 10.1054/ bjoc.2000.1899, available online at http://www.idealibrary.com on
http://www.bjcancer.com

### A population-based cohort study of HRT use and breast cancer in southern Sweden

**H Olsson[1,2], A Bladström[2], C Ingvar[3] and TR Möller[2]**

[1]Department of Oncology, University Hospital, Lund, S-22185, Sweden; [2]South Swedish Regional Tumour Registry, Lund, S-22185, Sweden; [3]Department of Surgery, University Hospital, Lund, S-22185, Sweden

**Summary** The overall tumour incidence and breast cancer incidence related to hormone replacement therapy (HRT) were followed in a population-based cohort of 29 508 women, aged 25–65 when interviewed in 1990–92. By the end of the follow up in December 1999, there were 226 611 person-years of observation. A total of 1145 malignant tumours were recorded (expected 1166.6; SIR = 0.98, 95% CI 0.93–1.04). There was a small excess of breast cancer with 434 observed and 387.69 expected (SIR = 1.12, 95% CI 1.02–1.23). Among about 3 663 ever users of HRT, there was no increase in overall tumour incidence (SIR = 0.98, 95% CI 0.86–1.12) but a significant excess of breast cancer (SIR = 1.35, 95% CI 1.09–1.64) compared with never users (SIR = 1.07, 95% CI 0.96–1.19). Breast cancer increased with increasing duration of use and for 48–120 months use the SIR was 1.92 (95% CI 1.32–2.70). There was no significant interaction with family history of breast cancer although an independent additive effect was suggested between HRT use and family history. In a Cox regression model time to breast cancer in relation to duration of HRT use was analysed adjusting for age at menarche, age at menopause, age at first full term pregnancy, parity and age at diagnosis. A significantly higher risk was seen for longer duration of HRT use compared with never users. No increased risk is seen in women beyond 5 years after stopping HRT. There was no interaction between previous use of oral contraceptives and later HRT use. © 2001 Cancer Research Campaign http://www.bjcancer.com

**Keywords**: hormone replacement therapy; tumour incidence; family history; present use; past use

Hormone replacement therapy (HRT) has become increasingly popular during the last decades. As yet the overall health benefit of HRT use is not known. An increased risk of breast and endometrial cancer has been seen (Kelsey and Whittemore, 1994), while there is a lower risk of osteoporosis (Genazzani and Gambacciani, 1999). A lower risk of cardiovascular disease and cognitive disorders as dementia has been hypothesized but is yet not supported by intervention studies (Genazzani and Gambacciani, 1999; Torgerson and Reid, 1999). The risk of endometrial cancer has been seen mainly for unopposed estrogen medication while the addition of progestins has normalised the risk (Pike and Ross, 2000). A higher risk of breast cancer has not been seen in all studies but recent studies, mainly reviewed in the collaborative meta-analysis, have demonstrated an increased risk (Collaborative Group on Hormonal Factors in Breast Cancer, 1997). Only a few cohort investigations exist (Mills et al, 1989; Hunt et al, 1990; Risch and Howe, 1994; Colditz et al, 1995; Persson, 1996; Lando et al, 1999; Schairer et al, 2000) and most studies find an increased incidence or mortality of breast cancer (Mills et al, 1989; Hunt et al, 1990; Risch and Howe, 1994; Colditz et al, 1995; Persson, 1996; Schairer et al, 2000). However there is still controversy about how high the risk is and whether it is transient or increasing with exposure and follow-up time. Recent studies have also suggested that preparations containing oestrogen alone do not increase the risk substantially while preparations containing both oestrogens and progestins do increase the risk (Magnusson et al, 1999; Ross et al, 2000; Schairer et al, 2000).

*Received 12 March 2001*
*Revised 25 April 2001*
*Accepted 2 May 2001*

*Correspondence to:* H Olsson

Some studies have suggested that the tumour biology of breast cancer associated with HRT use is favourable (Brinton et al, 1986; Magnusson et al, 1996; Holli et al, 1998; O'Connor et al, 1998) and that the prognosis is better than for other women diagnosed at a similar age (Jernström et al, 1999; Schairer et al, 1999). It has also been suggested that there is an increase in the incidence of lobular breast cancer but not of ductal breast carcinomas in the US and that this increase could be due to HRT use (Li et al, 2000a,b). The risk relationship requires further study with longer follow up, less possibility of recall bias, and with assessment of overall tumour incidence and mortality as well as the risk and mortality from other diseases. We have examined the risk for breast cancer in a cohort study in relation to time of exposure to HRT and family history of breast cancer.

### MATERIAL AND METHODS

40 000 women aged 25–65 years were randomly selected from the general population of the South Swedish Health Care Region and invited to take part in a standardized written interview of risk factors of malignant melanoma and breast cancer. No woman had a past history of malignancy. The interviews were performed between 1990–92. 1000 women from each birth year between age 25–65 were invited to participate in the study. About 74% of all women (*n* = 29 508) accepted to participate and participation was well balanced between the different age groups. In the different age groups 2803 women were finally included in age group 25–29, 3970 in age group 30–34, 3616 women in age group 35–39, 3639 in age group 40–44, 3647 in age group 45–49, 3658 in age group 50–54, 3495 in age group 55–59 and 4670 women in age group 60–65.

BJOC 01-1899 674-677 20/8/01 3:05 pm Page 675

The questionnaire included among other things questions about age at menarche, parity, age at first full-term pregnancy, age at menopause, type of menopause, oral contraceptive use (starting age, duration of use, brand use, age at last use), HRT use (starting age, duration of use, brand use, age at last use), family history of cancer/breast cancer, sun bathing habits, constitutional factors and alcohol and smoking habits.

With the help of the unique person identification number the vital status and the cancer incidence up to age 75 of these referents were then followed from the time of interview onward in the population based Census Registry, Cause of Death Registry and Swedish Cancer Registry (South Swedish Regional and National Swedish Tumour Registry). Each individual could have had more than one tumour registered. Observed versus expected number of tumours were then calculated using reference data from the southern health care region. The vital status was determined up to 1 January 2000. No subjects were lost to follow up. 5 women emmigrated and 589 women died during the follow-up time. Cause-specific standardized incidence ratios (SIRs) and 95% confidence intervals (CIs) were calculated. $P$ values were calculated by using the poisson distribution or the $\chi^2$ distribution if the expected values were greater than 10. The term 'significant' refers to $P < 0.05$. All tests are 2-tailed.

In Cox regression models the risk for breast cancer was modelled within the cohort while adjusting for possible confounding factors.

### RESULTS

At the end of the follow up in December 1999, the cohort constituted 226 611 person years. The median follow up time was 7.6 years. A total of 1145 malignant tumours were seen and 1166.6 were expected (SIR = 0.98, 95% CI 0.93–1.04). Slightly more breast cancer cases (434) were seen than expected (387.69) (SIR = 1.12, 95% CI 1.02–1.23). About 3663 women had ever used HRT. Ever use of HRT was associated with a significantly elevated risk for breast cancer (SIR = 1.35, 95% CI 1.09–1.64) compared with never use (SIR = 1.07, 95% CI 0.96–1.19). With increasing duration of use SIR increased (see Table 1) so a duration of HRT use between 48–120 months yielded a SIR = 1.92, 95% CI 1.32–2.70. There was no significant interaction between family history of breast cancer and HRT (see Table 2).

In a Cox regression model (Table 3) time to breast cancer in relation to duration of HRT use was analysed adjusting for age at menarche, age at menopause, age at first full term pregnancy, parity and age at diagnosis. A significantly higher risk was seen for longer duration of HRT use (> 48 months) compared with never users.

Restricting the regression analysis to women above 45 years of age and who had a natural menopause gave similar results (Table 4)

The risk of breast cancer was increased only for recent users while after more than 5 years of nonuse the risk was not significantly elevated (SIR = 1.38 versus SIR = 0.49).

There was no sign of interaction between a previous use of oral contraceptives and later HRT use as oral contraceptive use for more than 2 years followed by more than 4 years use of HRT yielded an SIR of 1.79 (95% CI 1.04–2.87).

### DISCUSSION

The main result of the present investigation is the finding of an increased risk of breast cancer after prolonged HRT use in a population-based cohort. After more than 4 years of exposure a SIR of 1.92 was noted. This risk is higher and appears after a shorter exposure than noted in most other investigations including the collaborative meta-analysis (Collaborative Group on Hormonal Factors in Breast Cancer, 1997). This could be due to the extensive use in south Sweden of combined hormone preparations, given that in some recent investigations a higher breast cancer risk has been seen for combined use than for oestrogen only preparations (Magnusson et al, 1999; Ross et al, 2000; Schairer et al, 2000). That oestrogen-only preparations were not frequently used is further substantiated by the finding of a low endometrial cancer risk. The risk of breast cancer in our study was independent of known risk factors of breast cancer and did not interact with a family history of breast cancer among first degree relatives. Although we did not analyse brand of HRT used, the predominant prescription in Sweden has been HRT containing both oestrogen and progestins given either as continuous or sequential therapy

**Table 1** Duration of hormone replacement therapy in relation to observed and expected number of breast cancer, SIR (95% CI), number of individuals and person years

| Duration (months) | OBS | SIR | 95% CI | *n* | Person years |
|---|---|---|---|---|---|
| 0, or never users | 345 | 1.06 | 0.95–1.18 | 26238 | 202 197 |
| 1–48 | 44 | 1.18 | 0.85–1.58 | 2005 | 15 049 |
| 48–120 | 33 | 1.92 | 1.32–2.70 | 851 | 6349 |
| 120+ | 12 | 1.36 | 0.70–2.37 | 414 | 3016 |

**Table 2** Hormone replacement therapy (HRT) in relation to a family history of breast cancer (first degree relative with breast cancer)

| HRT use (ever use) | OBS | SIR | 95% CI | *n* | Person years |
|---|---|---|---|---|---|
| Family history of breast cancer | 10 | 2.20 | 1.06–4.05 | 230 | 1723.5 |
| No family history of breast cancer | 84 | 1.31 | 1.04–1.62 | 3341 | 24953.2 |
| *Never HRT use* | | | | | |
| Family history of breast cancer | 35 | 1.77 | 1.24–2.47 | 1385 | 10 598 |
| No family history of breast cancer | 304 | 1.02 | 0.91–1.14 | 24 460 | 188 659 |

*© 2001 Cancer Research Campaign* 

BJOC 01-1899 674-677 20/8/01 3:05 pm Page 676

**Table 3** Cox regression analysis studying time to breast cancer in relation to HRT use, family history, age at first full term pregnancy, nulliparity and age at menarche in women experiencing menopause. Hazard ratios are adjusted for each factor simultaneously and year of diagnosis and menopausal age

| | Hazard ratio | 95% CI | *P* value |
|---|---|---|---|
| HRT use | | | |
| 0 months | 1.0 | | |
| 1–48 months | 1.36 | 0.98–1.90 | 0.07 |
| 48+ months | 1.80 | 1.27–2.56 | 0.001 |
| Family history of breast cancer | 1.86 | 1.33–2.58 | 0.001 |
| Age at first full term pregnancy | | | |
| >35 years | 1.89 | 1.15–3.09 | 0.01 |
| Nulliparity | 1.11 | 0.83–1.50 | 0.49 |
| Age at menarche | | | |
| >13 years of age | 0.96 | 0.78–1.18 | 0.69 |

**Table 4** Cox regression analysis studying time to breast cancer in relation to HRT use, family history, age at first full-term pregnancy, nulliparity and age at menarche in women experiencing menopause. Hazard ratios are adjusted for each factor simultaneously and year of diagnosis and menopausal age. Restricting the analysis to women with a natural menopause and with an age at interview above 45 years of age

| | Hazard ratio | 95% CI | *P* value |
|---|---|---|---|
| HRT use | | | |
| 0 months | 1.0 | | |
| 1–48 months | 1.20 | 0.86–1.67 | 0.28 |
| 48+ months | 1.78 | 1.26–2.51 | 0.001 |
| Family history of breast cancer | 1.82 | 1.30–2.54 | 0.0001 |
| Age at first full term pregnancy | | | |
| >35 years | 1.54 | 0.90–2.64 | 0.11 |
| Nulliparity | 1.35 | 0.99–1.83 | 0.06 |
| Age at menarche | | | |
| >13 years of age | 1.06 | 0.72–1.58 | 0.76 |

while monotherapy with oestrogens has been reserved for women who have had a prior hysterectomy. Whether 10 years or more of HRT use confers an even higher breast cancer risk can at present not be addressed using this cohort given that, as of yet, too few women have been exposed to HRT for such a duration.

In line with the data from the Collaborative Group on Hormonal Factors in Breast Cancer (Collaborative Group on Hormonal Factors in Breast Cancer, 1997), we found that recent HRT use conferred an increased risk of breast cancer while women who had not used HRT for at least 5 years prior to diagnosis were at no increased risk.

Our study can not confirm that HRT confers an especially high risk of breast cancer to women who have previously used oral contraceptives (Brinton et al, 1998).

It is interesting to note that although breast cancer incidence was increased no overall increase in cancer incidence was noted after HRT use. It thus seems that HRT use increases tumour incidence mainly for breast cancer, but this increase is counteracted by a lower incidence of other tumours.

Compared with another Swedish cohort investigation of HRT use the present cohort investigation has the advantage of retrieving the HRT information by direct interviews and not by prescriptions filled at pharmacies (Persson et al, 1997). The recall of the exposure was further aided by time calendar and charts of brands prescribed in Sweden. Furthermore the present cohort is population based and not limited to the use of certain pharmacies, hospitals or attending mammography units.

There is a possibility that the hazard ratio in the within cohort comparison is underestimated as women, who in the base-line questionnaire stated never use, may have become users of HRT during the follow-up time. The extent of such an underestimate will be assessed after completion of an on-going reinterview of the cohort. Further, the importance of different types of HRT will in a future study be addressed by comparing questionnaire data with hospital record information.

In conclusion our investigation confirms a rather high risk for breast cancer after at least 4 years of HRT use. The absolute risk seems to be independent of family history of breast cancer. The overall cancer incidence is not increased compared to the expected rate in HRT users. No increased risk is seen in women after 5 years of non-use.

### ACKNOWLEDGEMENTS

Supported by grants from the Swedish Cancer Society, the Medical Faculty of Lund University, CTRF, Lund University Hospital, and the Gunnar Nilsson Foundation.

### REFERENCES

Brinton LA, Hoover R and Fraumeni JF (1986) Menopausal oestrogens and breast cancer risk: an expanded case-control study. *Br J Cancer* **54**: 825–832

Brinton LA, Brogan DR, Coates RJ, Swanson CA, Potischman N and Stanford JL (1998) Breast cancer risk among women under 55 years of age by joint effects

 *© 2001 Cancer Research Campaign*

BJOC 01-1899 674-677 20/8/01 3:05 pm Page 677

of usage of oral contraceptives and hormone replacement therapy. *Menopause* **5**: 145–151

Colditz GA, Hankinson SE, Hunter DJ, Willett WC, Manson JE, Stampfer MJ, Hennekens C, Rosner B and Speizer FE (1995) The use of estrogens and progestins and the risk of breast cancer in postmenopausal women [see comments]. *N Engl J Med* **332**: 1589–1593

Collaborative Group on Hormonal Factors in Breast Cancer. (1997) Breast cancer and hormone replacement therapy: collaborative re-analysis of data from 49 epidemiological studies involving 51, 977 women with breast cancer and 107,283 women without breast cancer. *Lancet* **350**: 1047–1059

Genazzani A and Gambacciani M (1999) Hormone replacement therapy; the perspective for the 21st century. *Maturitas* **32**: 11–17

Holli K, Isola J and Cuzick J (1998) Low biologic aggressiveness in breast cancer in women using hormone replacement therapy. *J Clin Oncol* **16**: 3115–3120

Hunt K, Vessey M and McPherson K (1990) Mortality in a cohort of long-term users of hormone replacement therapy: an updated analysis. *Br J Obstet Gynaecol* **97**: 1080–1086

Jernström H, Frenander J, Fernö M and Olsson H (1999) Hormone replacement therapy before breast cancer diagnosis significantly reduces the overall death rate compared with never use among 984 breast cancer patients. *Br J Cancer* **80**(**9**): 1453–1458

Kelsey JL and Whittemore AS (1994) Epidemiology and primary prevention of cancers of the breast, endometrium, and ovary. A brief overview. *Ann Epidemiol* **4**: 89–95

Lando JF, Heck KE and Brett KM (1999) Hormone replacement therapy and breast cancer risk in a nationally representative cohort. *Am J Prev Med* **17**: 176–180

Li CI, Anderson BO, Porter P, Holt SK, Daling JR and Moe RE (2000a) Changing incidence rate of invasive lobular breast carcinoma among older women. *Cancer* **88**: 2561–2569

Li CI, Weiss NS, Stanford JL and Daling JR (2000b) Hormone replacement therapy in relation to risk of lobular and ductal breast carcinoma in middle-aged women. *Cancer* **88**: 2570–2577

Magnusson C, Holmberg L, Norden T, Lindgren A and Persson I (1996) Prognostic characteristics in breast cancers after hormone replacement therapy. *Breast Cancer Res Treat* **38**: 325–334

Magnusson C, Baron JA, Correia N, Bergstrom R, Adami HO and Persson I (1999) Breast-cancer risk following long-term oestrogen- and oestrogen-progestin-replacement therapy. *Int J Cancer* **81**: 339–344

Mills PK, Beeson WL, Phillips RL and Fraser GE (1989) Prospective study of exogenous hormone use and breast cancer in Seventh-day Adventists. *Cancer* **64**: 591–597

O'Connor IF, Shembekar MV and Shousha S (1998) Breast carcinoma developing in patients on hormone replacement therapy: a histological and immunohistological study. *J Clin Pathol* **51**: 935–938

Persson I (1996) Cancer risk in women receiving estrogen-progestin replacement therapy. *Maturitas* **23** (Suppl): S37–S45

Persson I, Bergkvist L, Lindgren C and Yuen J (1997) Hormone replacement therapy and major risk factors for reproductive cancers, osteoporosis, and cardiovascular diseases: evidence of confounding by exposure characteristics. *J Clin Epidemiol* **50**: 611–618

Pike MC and Ross RK (2000) Progestins and menopause: epidemiological studies of risks of endometrial and breast cancer [In Process Citation]. *Steroids* **65**: 659–664

Risch HA and Howe GR (1994) Menopausal hormone usage and breast cancer in Saskatchewan: a record-linkage cohort study. *Am J Epidemiol* **139**: 670–683

Ross RK, Paganini-Hill A, Wan PC and Pike MC (2000) Effect of hormone replacement therapy on breast cancer risk: estrogen versus estrogen plus progestin [see comments]. *J Natl Cancer Inst* **92**: 328–332

Schairer C, Gail M, Byrne C, Rosenberg PS, Sturgeon SR, Brinton LA and Hoover RN (1999) Estrogen replacement therapy and breast cancer survival in a large screening study. *J Natl Cancer Inst* **91**: 264–270

Schairer C, Lubin J, Troisi R, Sturgeon S, Brinton L and Hoover R (2000) Menopausal estrogen and estrogen-progestin replacement therapy and breast cancer risk [see comments]. *Jama* **283**: 485–491

Torgerson DJ and Reid DM (1999) The pharmacoeconomics of hormone replacement therapy. *Pharmacoeconomics* **16**: 9–16

© 2001 Cancer Research Campaign
